# The Protein Kinase Inhibitor Midostaurin Improves Functional Neurological Recovery and Attenuates Inflammatory Changes Following Traumatic Cervical Spinal Cord Injury

**DOI:** 10.3390/biom11070972

**Published:** 2021-07-01

**Authors:** Mohammad-Masoud Zavvarian, James Hong, Mohamad Khazaei, Jonathon Chon Teng Chio, Jian Wang, Anna Badner, Michael G. Fehlings

**Affiliations:** 1 Division of Genetics and Development, Krembil Brain Institute, University Health Network, Toronto, ON M5T 2S8, Canada; Mohammad.zavvarian@mail.utoronto.ca (M.-M.Z.); James.hong@live.com (J.H.); Mohammad.khazaei@uhnresearch.ca (M.K.); Jct.chio@mail.utoronto.ca (J.C.T.C.); Jian.wang@uhnresearch.ca (J.W.); Anna.badner@gmail.com (A.B.); 2 Institute of Medical Science, Faculty of Medicine, University of Toronto, Toronto, ON M5S 1A8, Canada; 3 Department of Surgery, Faculty of Medicine, University of Toronto, Toronto, ON M5T 1P5, Canada

**Keywords:** spinal cord injury, midostaurin, protein kinases, neuroprotection, neuroinflammation

## Abstract

Traumatic spinal cord injury (SCI) impairs neuronal function and introduces a complex cascade of secondary pathologies that limit recovery. Despite decades of preclinical and clinical research, there is a shortage of efficacious treatment options to modulate the secondary response to injury. Protein kinases are crucial signaling molecules that mediate the secondary SCI-induced cellular response and present promising therapeutic targets. The objective of this study was to examine the safety and efficacy of midostaurin—a clinically-approved multi-target protein kinase inhibitor—on cervical SCI pathogenesis. High-throughput analyses demonstrated that intraperitoneal midostaurin injection (25 mg/kg) in C6/7 injured Wistar rats altered the local inflammasome and downregulated adhesive and migratory genes at 24 h post-injury. Treated animals also exhibited enhanced recovery and restored coordination between forelimbs and hindlimbs after injury, indicating the synergistic impact of midostaurin and its dimethyl sulfoxide vehicle to improve functional recovery. Furthermore, histological analyses suggested improved tissue preservation and functionality in the treated animals during the chronic phase of injury. This study serves as a proof-of-concept experiment and demonstrates that systemic midostaurin administration is an effective strategy for mitigating cervical secondary SCI damage.

## 1. Introduction

Spinal cord injury (SCI) is a life-threatening and multifaceted condition that impairs the local neural circuitry responsible for both sensorimotor and autonomic functions [1]. The global age-standardized prevalence of SCI is, on average, 368 per 100,000, which accounts for an estimated annual 9.5 million years lived with disability (YLD) globally [2,3]. Despite recent clinical advances in rehabilitative and neuromodulatory treatments, SCI patients suffer from devastating consequences to their health, independence, and lifestyle [4,5]. Following trauma, the laceration, compression, and contusion of the spinal cord damages the local neuronal, glial, and vascular cells, which introduces toxic cellular debris and disrupts the vital vasculature network [6]. These events lead to a secondary pathology cascade, evident by early signs of hypoxia, microglial activation, reactive immune cell infiltration, and astrocytic response [7]. Over the course of the secondary injury, a non-neuronal lesion core characterized by cavitation and fibrotic scarring is formed at the injury epicenter [8]. The newly proliferated astrocytes, intermingled with inhibitory molecules, encapsulate this non-neuronal lesion core, marking a distinctive barrier to the spared perilesional zone in which neurons undergo injury-induced synaptic alterations and plasticity [9,10].

Neuroprotective treatments can potentially reduce the impact of the secondary injury on the spinal cord and improve functional recovery [11,12]. The currently approved neuroprotective treatment for SCI patients is an acute high-dosage methylprednisolone sodium succinate (MPSS) regimen aimed at suppressing the immune response to the injury [13]. The efficacy of MPSS is limited, as it only provides modest improvements at the expense of an increased risk of pneumonia and infection [14,15]. Hence, there is a need for a potent and targeted neuroprotective treatment to alleviate the SCI-induced secondary response.

Protein kinases are a class of globular enzymes involved in various cell signaling pathways that facilitate the induced cellular processes after traumatic SCI [16]. There are more than 450 protein kinases in the human genome, and they play critical roles in many essential cellular pathways [17,18]. Through the reversible phosphorylation of downstream substrates, these kinases are capable of altering gene expression patterns in affected cell types. Aberrant kinase activity is believed to be implicated in the progression of secondary SCI [19,20]. Many small-molecule kinase inhibitors (with varying degrees of specificity) have been investigated to reduce neuropathic pain, axonal disruption, and immunoreactivity after SCI [21,22]. These small-molecule kinase inhibitors have broad efficacy and excellent tissue-penetrance [18]. Notably, we previously conducted several preclinical and clinical studies examining the neuroprotective effect of riluzole, a sodium channel blocker that can also directly inhibit protein kinases [23,24]. Riluzole improves functional recovery in rat cervical injuries [25,26], and is currently being examined in a phase 2/3 clinical trial (ClinicalTrials.gov Identifier: NCT01597518).

Midostaurin (also referred to as PKC412 or Rydapt) is a clinically approved small-molecule inhibitor of multiple protein kinases that is currently used for acute myeloid leukemia (AML) [27,28], mast cell leukemia, and systemic mastocytosis [29,30]. Midostaurin is capable of spontaneously entering the central nervous system (CNS), and it can present mild side effects, such as febrile neutropenia, nausea, and headaches. These symptoms are known to be manageable and predictable in their onset and reversible upon the cessation of the drug [30,31,32,33]. As a staurosporine-derived inhibitor, midostaurin competitively binds to the ATP binding site, rendering protein kinases ineffective [34]. Midostaurin is metabolized in the liver by the enzyme CYP3A4 and has been shown to interact with CYP3A4 inhibitors and inducers [27,34]. In vitro assays have demonstrated that midostaurin (half-life of 21 h) and its major active metabolites CGP62221 (half-life of 32 h) and CGP52421 (half-life of 482 h) can inhibit the activity of several protein kinases including FMS-like tyrosine kinase 3 (FLT3), KIT, platelet-derived growth factor receptor alpha/beta (PDGFRα/β), vascular endothelial growth factor receptor 2 (VEGFR2), and protein kinase C (PKC) [35,36]. The reduced activity of these protein kinases has been shown to decrease the phosphorylation of downstream molecules, such as STAT5 and STAT3 [37,38].

The purpose of this study was to examine, for the first time, the potential role of the protein kinase inhibitor midostaurin in attenuating the early secondary pathogenesis following traumatic cervical SCI. Using inflammatory, transcriptional, histological, and neurobehavioral readouts, we report that midostaurin treatment is associated with a significant beneficial impact in a translationally relevant model of cervical SCI. Due to the vast diversity of staurosporine-derived inhibitors with varying specificities toward unique protein kinases [39], the present study is a crucial first step for exploring the therapeutic role of kinomic alterations in cervical SCI [40].

## 2. Materials and Methods

### 2.1. Animal Care and SCI

All animal experiments were performed on inbred female Wistar rats weighing between 230 and 280 g and aged between 12 and 13 weeks old (obtained from Charles River Laboratories, Wilmington, MA, USA, www.criver.com, accessed on 29 June 2021). Injured rats underwent a clip compression injury (1-min, 23.6 g, 1 mm in width) after laminectomy at the cervical region (C6/7; Supplementary Figure S1). Sham rats followed the same laminectomy procedure without undergoing clip-compression injury. All animal experiments were in compliance with the animal care committee at the University Health Network (UHN) and the Canadian Council on Animal Care. For the clip compression injury operation, 5% inhalant isoflurane in O_2_-carrying gas was used to anesthetize the rats, and 2% isoflurane was used to maintain anesthesia throughout the surgeries. Immediately post-surgery and before anesthesia emergence, the rats were subcutaneously administered with 0.05 mg/kg of buprenorphine and 5 mL of saline. Depending on the cohort, the rats were also intraperitoneally injected with either midostaurin (25 mg/kg) or vehicle control. Midostaurin was purchased from Selleckchem (Cat#: S8064; Burlington, Ontario, ON, Canada, www.selleckchem.com, accessed on 29 June 2021). The midostaurin solution was prepared by serial dilution in 10% *v/v* DMSO in saline to a final concentration of 2 mg/mL and mixed thoroughly. The vehicle solution followed the same procedure without the midostaurin addition. The solution was prepared on the day of administration and stored at 4 °C prior to injection.

### 2.2. Spleen Mass

All animals were weighed prior to the SCI operation, then weekly during neurobehavioral assessments, and ultimately prior to sacrifice. Spleens were collected prior to transcardial perfusion at 1-day and 56-days post-injury. The spleens were individually weighed and normalized to the time-matched total body mass.

### 2.3. RNA and Protein Extraction

Animals were sacrificed at 1-day post-injury, and the extracted injured spinal cords were prepared for transcriptional and protein analyses. To this aim, the rats were anesthetized with 5% inhalant isoflurane in O_2_-carrying gas. Following transcardial perfusion with 250 mL of a phosphate buffer solution (PBS), the injury epicenter spanning 1 cm in length (5 mm rostral and caudal from the injury site) was extracted and snap-frozen in liquid nitrogen. RNA and protein were extracted using the mirVana^TM^ PARIS^TM^ Kit (Cat#: AM1556; Toronto, ON, Canada, www.uoftmedstore.com, accessed on 29 June 2021) and stored at −80 °C. The total protein concentration was measured using the Micro BCA™ Protein Assay Kit (Cat#: 23235; Toronto, ON, Canada, www.uoftmedstore.com, accessed on 29 June 2021), and the total RNA concentration was measured using NanoDrop spectrophotometry (ND-1000, Wilmington, DE, USA).

### 2.4. RT-qPCR

The extracted RNA was reverse transcribed into cDNA using Bioline SensiFAST^TM^ cDNA Synthesis Kit (Cat#: BIO-65053, Toronto, ON, Canada, www.uoftmedstore.com, accessed on 29 June 2021) and diluted to a concentration of 1 ng/µL. Bioline SensiFAST™ Probe Hi-ROX (Cat#: BIO-82020, Toronto, ON, Canada, www.uoftmedstore.com, accessed on 29 June 2021) was mixed with TaqMan primers (10:1) and added to cDNA (4.5:5.5). Primers were obtained from Applied Biosystems (Foster City, CA, USA, www.thermofisher.com, accessed on 29 June 2021) and are listed in Supplementary Table S3. PPIA was used as the endogenous control. Each sample was examined in triplicate and followed 40 cycles. Subsequent analysis was performed with 2^−ΔΔCT^ (relative quantification) recordings using Applied Biosystems SDS RQ Manager (version 2.1).

### 2.5. Western Blotting

The samples were separated using sodium dodecyl sulphate–polyacrylamide gel electrophoresis (SDS-PAGE) for 60 min (Bio-Rad Precast Gradient gel, Mississauga, ON, Canada) and transferred into a nitrocellulose membrane. The total protein was visualized with a Ponceau S solution, and 3% milk was used to block the membrane. Subsequently, the membrane was incubated with primary antibodies overnight at 4 °C and washed with Tris Buffered Saline + Tween^®^ 20 (TBST) afterward (triple wash, 10 min). The primary antibodies included p-GSK3β (Cat#: 9336S), GSK3β (Cat#: 9315), p-STAT3 (Cat#: 9131S), and STAT3 (Cat#: 9139S), all of which were obtained from Cell Signaling (1:250 antibody in 3% milk ratio; Supplementary Table S1). Molecular weights were determined according to the Sigma-Aldrich BLUeye Prestained Protein Ladder. The membranes were subsequently incubated with secondary horseradish peroxidase (HRP) antibody at room temperature (1 h) and washed again with TBST (triple wash, 10 min; Supplementary Table S1). The membranes were probed by Bio-Rad Chemidoc^TM^ and analyzed with the Bio-Rad Image Lab^TM^ software (Version 6.0). Band densitometry was normalized to the total protein using Ponceau S staining [41,42,43,44,45]. Additionally, the sham group was used as a biological control to normalize the results. A logarithmic scale was used to illustrate up- or down-regulation compared to the shams with an intuitive positive or negative direction. Phosphorylated GSK3β and STAT3 represent the p-GSK3β and p-STAT3 band volumes, respectively, normalized to Ponceau S total protein staining. GSK3β and STAT3 phosphorylation represent the levels of phosphorylated GSK3β and STAT3 normalized to pan-GSK3β and pan-STAT3 levels, respectively.

### 2.6. Multiplex Luminex Assay

200 µg of the isolated protein (concentration determined by the Micro BCA™ Protein Assay Kit) was diluted to the total volume of 60 μL PBS. The samples were sent to Eve Technologies (Calgary, AB, Canada, www.evetechnologies.com, accessed on 29 June 2021) for Rat Cytokine Array/Chemokine Array 27 Plex (RD27)^TM^ analysis. The protein concentration (pg/mL) was calculated based on a standard curve for: eotaxin, EGF, fractalkine, IFN-γ, IL-1α, IL-1β, IL-2, IL-4, IL-5, IL-6, IL-10, IL-12(p70), IL-13, IL-17A, IL-18, IP-10, GRO/KC, TNF-α, G-CSF, GM-CSF, MCP-1, Leptin, LIX, MIP-1α, MIP-2, RANTES, and VEGF.

### 2.7. Neurobehavioral Assessments

Neurobehavioral assessments were performed weekly by blinded examiners. Forelimb motor function was scored using the forelimb grip strength measured with a grip strength meter (SDI Grip strength system, model DFM-10, San Diego Instruments, San Diego, California). Trunk stability was evaluated by inclined plane test [46]. At 8 weeks, the CatWalk^TM^ multivariate system (Noldus, Version 7.1) was used to assess paw and limb function during steady speed locomotion. In addition, a blinded observer scored the hindlimb function according to the Basso–Beattie–Bresnahan scale (BBB) [47].

### 2.8. Ultrasonography

The syrinx size in the spinal cord was assessed by pre-sacrificial echography with high-resolution ultrasound done using a 44 MHz probe (Vevo 770, VisualSonics, Toronto, ON, Canada), as previously described [48]. The 3D recordings were saved into the DICOM image format and analyzed using the TrakEM2 plugin within Fiji to quantify the cavitation volume.

### 2.9. Histomorphometric Analysis

Animals were sacrificed using transcardial perfusion (infused with 250 mL of PBS and 120 mL of 4% paraformaldehyde). Spinal cords spanning 1 cm around the lesion core (5 mm rostral and caudal from the injury site) were isolated and placed in post-fix using 10% sucrose in a 4% paraformaldehyde solution for 5 h. Samples were cryoprotected in 20% sucrose and PBS for 48 h. The cords were sectioned in Epredia™ M1 embedding matrix in 30 μm slices and stored at −80 °C. The sectioned spinal cords were stained with Luxol fast blue (LFB), as well as hematoxylin and eosin (H&E) to quantify the lesion and gray/white matter tissue every 480 μm, as previously described [49].

### 2.10. Immunohistochemistry

A blocking solution (containing 5% milk, 1% bovine serum albumin, and 0.03% Triton X-100 diluted in 1× PBS) was applied to the previously sectioned spinal cords. Anti-GAD67 primary mouse antibody (Cat#: MAB5406; 1:500 concentration in blocking buffer) and anti-NEUN antibody (Cat#: ABN78; 1:500 concentration in blocking buffer) were applied overnight at 4 °C (Supplementary Table S1). The fluorophore-conjugated secondary antibodies (Cat#: A-21235 and A-11034; 1:500 concentration in blocking buffer) were applied for 90 min at room temperature (Supplementary Table S1). A 1× PBS solution was used to wash the cords three times during the staining process to minimize noise. The slides were imaged using a Nikon C2+ confocal laser microscope. The GAD67 images were quantified by image thresholding in Fiji, and the % area of positive GAD67 staining was recorded [50]. All images were analyzed in Fiji by a blinded examiner.

### 2.11. Statistical and Bioinformatics Analysis

All statistical analyses were performed and visualized on GraphPad Prism 7. All measurements were examined for normality and homoscedasticity using the Shapiro–Wilk test. The comparisons made between two groups were assessed using a *t*-test, and the false discovery rate (FDR) according to the two-stage linear set-up procedure of Benjamini, Krieger, and Yekutieli was used to account for multiple testing in transcriptional and multiplex Luminex assay analyses. Heatmaps with hierarchical clustering based on cosine similarity were created in Morpheus (Broad Institute, Cambridge, MA, USA, https://software.broadinstitute.org/morpheus accessed on 29 June 2021). ANOVA with Holm–Sidak for multiple comparisons was used to compare more than two groups. For non-parametric analysis, a Kruskal–Wallis ANOVA was performed with Dunn’s multiple comparison test. Grouped analyses were investigated with two-way ANOVA using FDR to account for multiple comparisons according to the two-stage linear set-up procedure of Benjamini, Krieger, and Yekutieli, which avoids the stringent exclusion of potentially significant comparisons.

## 3. Results

### 3.1. Midostaurin Alters the Local Inflammatory Response

**Figure 1:**
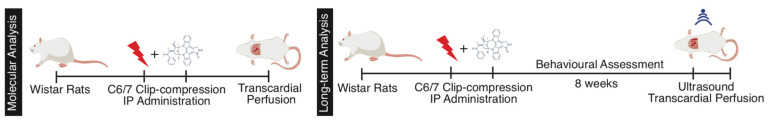
Schematic overview of experimental paradigms to assess the impact of acute intraperitoneal midostaurin administration after cervical SCI. In phase 1, the molecular impact of midostaurin at 24 h post-SCI was assessed using the multiplex Luminex assay and RT-qPCR. This phase consisted of four cohorts: C6/7 laminectomized sham (*n* = 6), C6/7 SCI control (*n* = 5), 0 mg/kg midostaurin (*n* = 6), and 25 mg/kg midostaurin (*n* = 6). In phase 2, the impact of midostaurin on functional recovery was assessed using four cohorts (*n* = 5 per cohort): C6/7 laminectomized sham, SCI control, 0 mg/kg midostaurin, and 25 mg/kg midostaurin.

**Figure 2:**
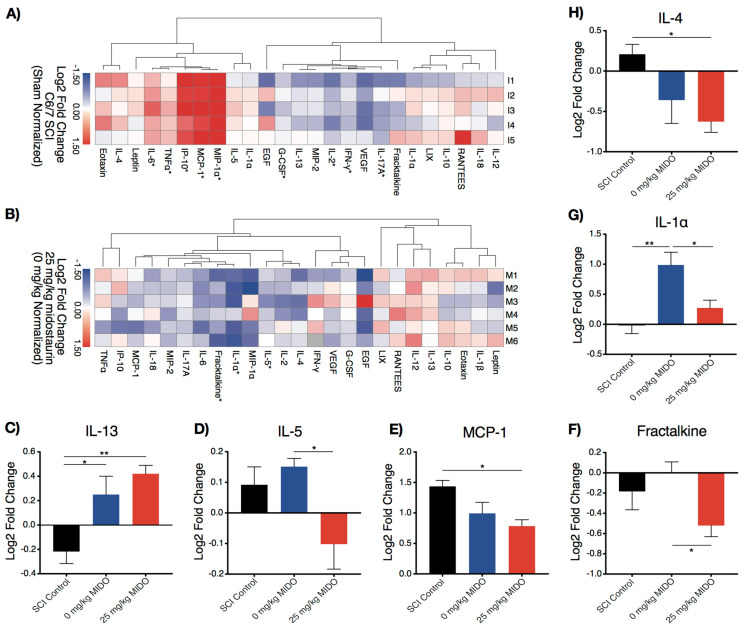
The local inflammatory response at 24 h post-operation in the laminectomized sham (*n* = 6), C6/7 SCI control (*n* = 5), 0 mg/kg midostaurin (*n* = 6), and 25 mg/kg midostaurin (*n* = 6) cohorts. (**A**,**B**) Hierarchical clustering was performed based on cosine similarity. Statistically significant changes are illustrated with an asterisk (*), as determined by a t-test using FDR according to the two-stage linear set-up procedure of Benjamini, Krieger, and Yekutieli to correct for multiple comparisons. (**A**) Heat map illustrates log_2_ fold change in the C6/7 SCI control cohort relative to shams. (**B**) Heat map illustrates log_2_ fold change in the 25 mg/kg midostaurin cohort compared to the 0 mg/kg midostaurin cohort. (**C**–**H**) Statistically significant expressions in the 25 mg/kg midostaurin cohort compared to either the 0 mg/kg midostaurin or C6/7 SCI control cohorts (determined by one-way ANOVA using Holm–Sidak for multiple comparisons; demonstrated in log_2_ sham-normalized fold change ± SEM; *: *p* < 0.05; **: *p* < 0.01).

The local molecular impact of midostaurin was examined at 24 h after cervical SCI (Figure 1). Western blotting revealed lower levels of phosphorylated GSK3β and STAT3 in the midostaurin-treated group (Supplementary Figure S2). These are multirole signal transducer molecules downstream of protein kinases that validate the impact of midostaurin on local enzymatic activity. A subsequent high-throughput multiplex Luminex assay demonstrated changes in the local inflammasome after midostaurin administration. Following C6/7 clip-compression injury, the levels of IL-6, TNFα, IP-10, MCP-1, MIP-1α, G-CSF, IL-2, IFN-γ, and IL-17A were significantly altered at the injury epicenter compared to uninjured shams (Figure 2A). The 0 mg/kg midostaurin vehicle control cohort demonstrated elevated levels of IL-1α (1.01 ± 0.24 log_2_ fold change, adjusted *p* < 0.01) and IL-13 (0.47 ± 0.16, adjusted *p:* 0.03) compared to the C6/7 SCI control. The administration of 25 mg/kg midostaurin significantly reduced the amount of IL-1α (-0.72 ± 0.23 log_2_ fold change, adjusted *p:* 0.02), fractalkine (−0.52 ± 0.18 log_2_ fold change, adjusted *p:* 0.03), and IL-5 (−0.25 ± 0.08 log_2_ fold change, adjusted *p:* 0.03) (Figure 2D,F,G). Compared to the C6/7 SCI control cohort, 25 mg/kg midostaurin significantly reduced the level of IL-4 (−0.84 ± 0.29 log_2_ fold change, adjusted *p*: 0.04) and MCP-1 (−0.65 ± 0.20 log_2_ fold change, adjusted *p:* 0.02) but increased the level of IL-13 (0.64 ± 0.16 log_2_ fold change, adjusted *p:* 0.005). These affected cytokines are key mediators of the inflammatory response after injury (Supplementary Table S2). Hierarchical clustering suggests a close association of IL-1α and fractalkine due to midostaurin administration (Figure 2B). Furthermore, no statistically significant splenic or body mass changes were detected at 24 h post-SCI.

### 3.2. Midostaurin Reduces the Expression of Trans-Endothelial Migratory Genes

**Figure 3:**
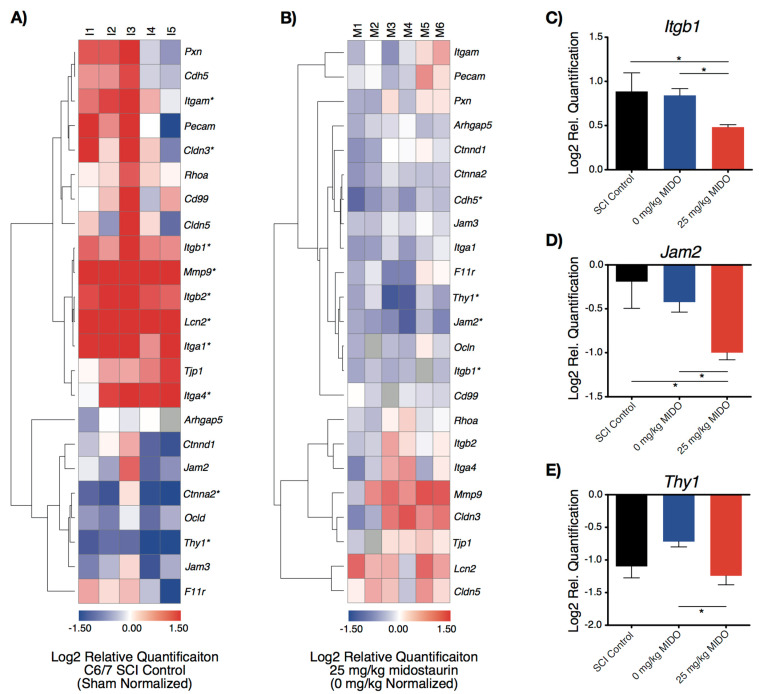
Expression of trans-endothelial migratory genes (KEGG Pathway: rno04670) at 24 h post operation in the laminectomized sham (*n* = 6), C6/7 SCI control (*n* = 5), 0 mg/kg midostaurin (*n* = 6), and 25 mg/kg midostaurin (*n* = 6) cohorts. (**A**,**B**) Hierarchical clustering was based on cosine similarity. Statistically significant changes are illustrated with an asterisk (*), as determined by a t-test using FDR according to the two-stage linear set-up procedure of Benjamini, Krieger, and Yekutieli to correct for multiple comparisons. (**A**) Heat map illustrates log_2_ fold change in the C6/7 SCI control cohort relative to uninjured shams. (**B**) Heat map illustrates log_2_ fold change in the 25 mg/kg midostaurin-treated SCI cohort compared to the 0 mg/kg midostaurin SCI group. (**C**–**E**) Statistically significant expressions in 25 mg/kg midostaurin-treated SCI animals compared to 0 mg/kg midostaurin-treated SCI animals for genes involved in the adhesion of immune cells to the spinal endothelial cells (determined by one-way ANOVA using Holm–Sidak for multiple comparisons; demonstrated in log_2_ sham-normalized relative quantification ± SEM; *: *p* < 0.05).

The local inflammatory response following cervical SCI requires the infiltration of reactive immune cells to the site of injury. In order to further investigate the therapeutic role of midostaurin on injury-induced inflammation, the effects of midostaurin on the transcription of trans-endothelial migratory (TEM) genes were examined using selective RT-qPCR. The selected genes were chosen according to the Kyoto Encyclopedia of Genes and Genomes (KEGG) and are involved in the TEM pathway (KEGG Pathway: rno04670). Hierarchical clustering suggested a differential expression pattern at the injury epicenter due to both the utilized injury model, as well as midostaurin administration. Out of the 23 analyzed genes, 10 genes were differentially expressed in the C6/7 SCI control group compared to the uninjured shams. Specifically, the injury model significantly altered the expression of *Lcn2 Mmp9, Itga1, Itgb2, Thy1, Itga4, Cldn3, Ctnna2, Itgb1,* and *Itgam* (Figure 3A). The administration of 25 mg/kg midostaurin reduced the expression of genes involved in migratory and adhesive function, such as *Jam2* (-0.58 ± 0.24 log_2_RQ, adjusted *p:* 0.03), *Thy1* (0.52 ± 0.18 log_2_RQ, adjusted *p:* 0.04), and *Itgb1* (8.47 mean rank difference, adjusted *p:* 0.048) at 24 h post-SCI (Figure 3B,C). Additionally, there was a notable decrease in the expression of *Cdh5* in 25 mg/kg midostaurin compared to the 0 mg/kg midostaurin cohort (Figure 3B).

### 3.3. Midostaurin and DMSO Act Synergistically to Improve Functional Recovery

**Figure 4:**
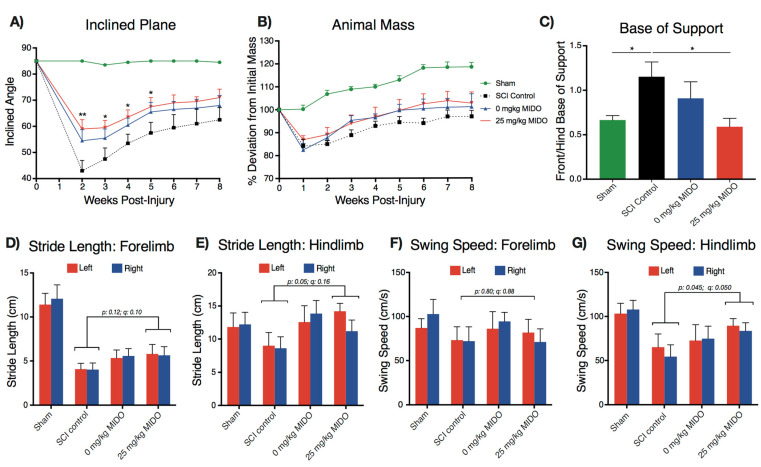
Acute administration of midostaurin improves functional recovery after cervical SCI (*n* = 5 per cohort). (**A**) Inclined plane assessment; the 25 mg/kg midostaurin-treated group had significantly enhanced functional recovery compared to the SCI control. (**B**) Animal mass recorded weekly suggested an improved total body mass recovery in both the 0 mg/kg midostaurin and 25 mg/kg midostaurin cohorts compared to the SCI control. (**A**,**B**) Time-course data analyzed with two-way ANOVA using FDR according to the two-stage linear set-up procedure of Benjamini, Krieger, and Yekutieli to correct for multiple comparisons. Statistically significant changes are illustrated with an asterisk (*). (**C**) Improved hindlimb/forelimb base of support in the 25 mg/kg midostaurin group compared to the SCI control. The comparison was made by one-way ANOVA using Holm–Sidak for multiple comparisons (*: *p* < 0.05; **: *p* < 0.01). (**D**–**G**) CatWalk^TM^ analysis demonstrated enhanced recovery in stride length and swing speed after DMSO and midostaurin administration (two-way ANOVA using FDR according to the two-stage linear set-up procedure of Benjamini, Krieger, and Yekutieli to correct for multiple comparisons). All data are presented as mean ± SEM.

A randomized long-term behavioral assessment was conducted to examine the recovery and well-being of animals following injury (Figure 1). The assessment included four cohorts (*n* = 5): laminectomized shams, C6/7 SCI control animals, injured animals receiving 0 mg/kg midostaurin, and injured animals receiving 25 mg/kg midostaurin. The inclined plane assessment [46] displayed significantly enhanced recovery in the 25 mg/kg midostaurin cohort compared to the C6/7 SCI control animals (Figure 4A). This improved inclined plane assessment was largely due to early recovery (16° ± 4.9°, *p* < 0.01, and *q* < 0.01 at 2 weeks post-SCI). The BBB [47] scoring reached statistical significance for improved hindlimb function at 6 weeks post-injury in the 25 mg/kg midostaurin cohort compared to the C6/7 SCI control animals (Supplementary Figure S3A; 3.2 ± 1.18 BBB score, *p*: 0.01, and *q*: 0.01). However, DMSO exerted a modest neuroprotective effect, as evident in the improved recovery in the 0 mg/kg midostaurin cohort compared to the SCI control (Figure 4A–G). Interestingly, both the 0 mg/kg midostaurin and 25 mg/kg midostaurin cohorts demonstrated a modest recovery of total body mass after injury (Figure 4B). However, the post-sacrifice splenic mass was not altered in any of the injured cohorts (Supplementary Figure S3B). Furthermore, no statistically significant differences in forelimb grip-strength were observed in either the 0 mg/kg midostaurin or 25 mg/kg midostaurin groups compared to the SCI control cohort (Supplementary Figure S3C).

The CatWalk^TM^ gait assessment suggested a differential locomotor pattern between the uninjured sham, SCI control, and midostaurin-treated animals. Previously, we demonstrated reduced swing speed and stride length following cervical SCI [51]. The present study observed similar SCI-induced locomotor alterations in both hindlimbs and forelimbs (Figure 4D−G). The animals that received 25 mg/kg midostaurin exhibited improved hindlimb stride length (3.89 cm ± 1.90 cm, *p*: 0.05, and *q*: 0.16) and swing speed (26.78 s ± 12.85 s, *p*: 0.045, and *q*: 0.05) compared to the SCI control (Figure 4). Strikingly, the 25 mg/kg midostaurin cohort demonstrated a restored ratio of forelimb to hindlimb base of support (Figure 4C; 0.56 ± 0.19 forelimb/hindlimb, adjusted *p:* 0.03).

### 3.4. Histomorphometric Analysis Reveals Improved Tissue Preservation

**Figure 5:**
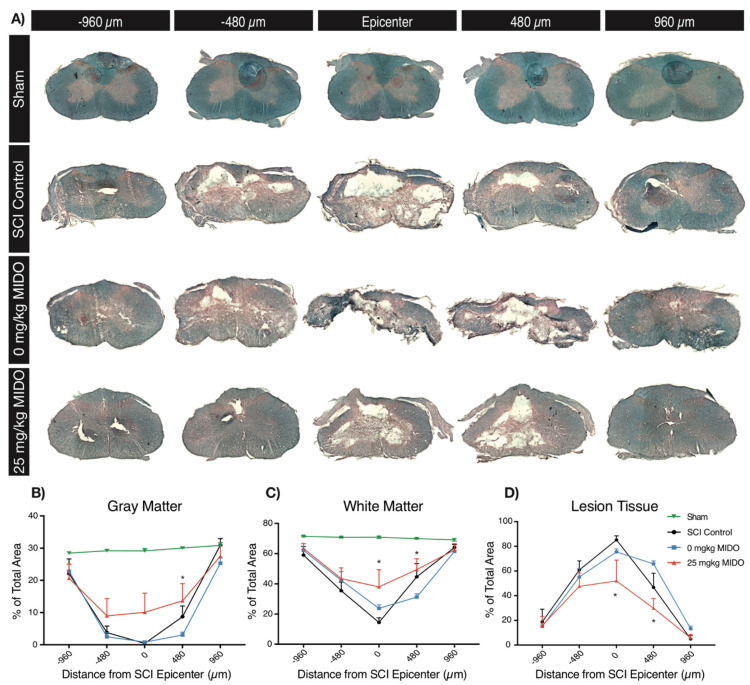
LFB and H&E staining revealed increased tissue preservation in both gray and white matter at 8 weeks post-SCI (*n* = 5 per cohort). (**A**) Representative images show the injury epicenter, as well as 480 and 960 µm rostral and caudal to the injury epicenter in the sham, SCI control, 0 mg/kg midostaurin, and 25 mg/kg midostaurin cohorts. (**B**) There was a significant difference in gray matter volume in all injured groups compared uninjured shams. Animals receiving 25 mg/kg midostaurin demonstrated improved gray matter preservation. (**C**) The injury reduced white matter volume, and 25 mg/kg midostaurin enhanced white matter preservation. (**D**) The lesioned tissue size was reduced in the 25 mg/kg midostaurin-treated group. Data are presented as mean ± SEM values and were assessed using two-way ANOVA with FDR according to the two-stage linear set-up procedure of Benjamini, Krieger, and Yekutieli to correct for multiple comparisons (*: *p* < 0.05).

The gray and white matter ratio and the lesion tissue volume were quantified using LFB and H&E staining to further explore the impact of midostaurin on tissue preservation at 8 weeks post-SCI. As expected, no lesion tissue was present in the uninjured shams (Figure 5A). All injured groups exhibited a lower reduction in the gray and white matter volume compared to the uninjured shams. The lesion volume was significantly lower in the 25 mg/kg midostaurin-treated group compared to the 0 mg/kg midostaurin group at the injury epicenter (Figure 5D; 23.52% ± 10.56%, *p*: 0.03, and *q*: 0.02), as well as 480 µm caudal to the injury site (Figure 5D; 35.91% ± 9.96%, *p*: 0.007, and *q*: 0.002). Similarly, the administration of 25 mg/kg midostaurin significantly enhanced gray and white matter preservation at 480 µm caudal to the injury site (Figure 5B,C). Additionally, the white matter preservation was significantly higher at the epicenter in the 25 mg/kg midostaurin group compared to the SCI controls (23.58% ± 8.03%, *p*: 0.005, and *q*: 0.01).

**Figure 6:**
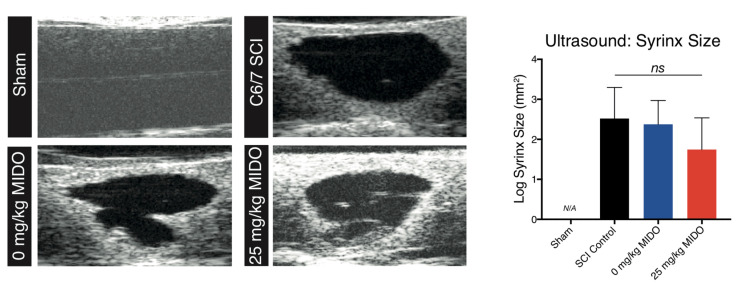
Ultrasonographical imaging of live spinal cord tissue revealed no significant changes in syrinx volume in the 25 mg/kg midostaurin group. Representative in vivo very high-resolution ultrasound imaging in B-Mode (11 × 7 mm) shown in sham, SCI control, 0 mg/kg midostaurin, and 25 mg/kg midostaurin cohorts (8 weeks post-surgery). No cavitation was visible in the sham animals. The graph represents ultrasonographical volume quantification (*n* = 5 per cohort), as assessed with one-way ANOVA. Data are presented as mean ± SEM values. *ns*: not significant.

High-resolution ultrasonography computed the syrinx size at 8 weeks post-injury prior to animal sacrifice [48]. Consistent with our previous results [52], cavitation was absent in the non-injured sham group (Figure 6). However, no observable difference in cavitation was detectable in the injured groups (Figure 6; one-way ANOVA, *p*: 0.73).

### 3.5. Immunohistological Examinations Reveal Restored GAD67 Level

**Figure 7:**
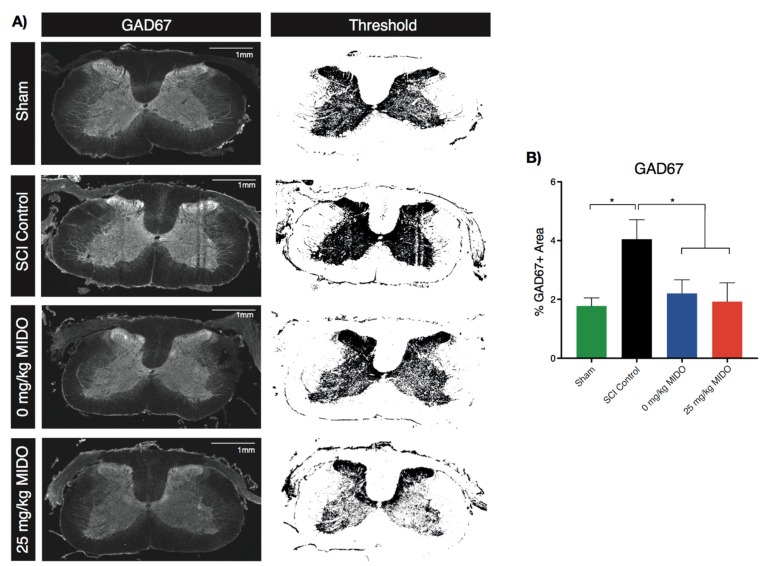
Restored GAD67 availability in treated animals at 960 μm rostral to the C6/7 epicenter. (**A**) Representative GAD67 staining under 2X confocal imaging illustrated in the C6/7 laminectomized sham, SCI control, 0 mg/kg midostaurin, and 25 mg/kg midostaurin cohorts. (**B**) GAD67 quantification suggested a significant increase in the availability of GAD67 after injury. GAD67 returned to the normal stage in both the 0 mg/kg midostaurin and 25 mg/kg midostaurin cohorts. Data are presented as mean ± SEM values and were assessed using one-way ANOVA using Holm–Sidak for multiple comparisons. *: *p* < 0.05.

To further investigate the molecular mechanisms behind the improved forelimb and hindlimb coordination, we stained for glutamate decarboxylase (GAD67) at the cervical level caudal to the injury site. GAD67 is responsible for the conversion of glutamate to GABA in neurons and is crucial for the maintenance of excitatory and inhibitory signals in the spinal cord. The results demonstrated restored GAD67 in both the 0 mg/kg midostaurin (−1.85 ± 0.75% GAD67+ area, adjusted *p:* 0.03) and 25 mg/kg midostaurin (−2.13 ± 0.75% GAD67+ area, adjusted *p:* 0.03) cohorts compared to the SCI control (Figure 7; Supplementary Figure S4).

## 4. Discussion

This is the first study to demonstrate the efficacy and safety of midostaurin as a neuroprotective therapeutic for traumatic cervical SCI. This is a crucial first step for repurposing midostaurin or other staurosporine-derived inhibitors for CNS injuries. The present study has demonstrated the ability of midostaurin to attenuate the local inflammasome and downregulate the expression of adhesive genes in the injured spinal cord, in addition to improving long-term neurobehavioral and histological assessments at 8 weeks post-SCI. These observed outcomes were consistent with the previous literature on the impact of midostaurin on inflammatory markers [53,54]. In addition, the results echoed the previously tested pharmacological agents capable of kinomic interventions, such as riluzole. However, important caveats remain to be addressed. Notably, DMSO masks the impacts of midostaurin on behavioral improvements. Thus, the observed molecular changes and improvement in forelimb and hindlimb coordination in treated animals compared to the SCI control warrant closer consideration regarding the applicability of early neuroprotectors to restore functional neuronal connectivity.

### 4.1. Early Molecular Response to Midostaurin

Our lab and others have previously profiled SCI-induced secondary damage in the context of vascular-mediated and inflammatory-mediated pathogenesis [55,56]. In rodent models of clip-compression injury, the peak of secondary vascular pathology has been shown to be at 24 h post-SCI, and targeting this phase can have a lasting neuroprotective effect on tissue in the chronic phase [55,56]. The present study thus aimed to assess the early molecular response to midostaurin administration at 24 h post-SCI and to subsequently examine the long-term impact of this neuroprotective treatment.

GSK3β is a well-known and ubiquitous downstream target of protein kinases, and a change in its phosphorylation is indicative of midostaurin penetrance to the injury epicenter [57]. GSK3β contains three phosphorylation sites at serine 9, threonine 43, and serine 389. The serine 9 residue is actively phosphorylated by various protein kinases and regulates important cellular processes such as synaptic plasticity [58,59,60]. STAT3 is similarly phosphorylated by protein kinases. STAT3 can be phosphorylated in the presence of G-CSF and MCP-1/CCR2, thus suggesting its involvement in propagating inflammation and the immune response [61,62]. Furthermore, STAT3 knockout reduces astrocyte migration and proliferation, as the astroglial border is formed via the STAT3-dependent interaction of newly proliferated astrocytes to confine the inflammatory and fibrotic cells after SCI [63,64]. Taken together, the attenuated kinomic activity, as evidenced by reduced phosphorylated STAT3 and GSK3β, can induce molecular cascades in neuronal, glial, and vascular cells.

Junctional and adhesive proteins are important for the integrity and permeability of the blood–spinal cord barrier (BSCB). The adhesive proteins along with chemotactic factors are crucial for the recruitment of blood-borne immune cells across the BSCB [65,66,67]. The present study demonstrated the reduced expression of adhesive and migratory genes, including *Jam2*, *Thy1*, and *Itgb1,* in the 25 mg/kg midostaurin cohort. *Jam2* is a member of junctional adhesion molecules expressed by endothelial cells and regulates vascular permeability and leukocyte transmigration across endothelial-cell surfaces [67]. *Itgb1* codes for integrin beta 1 and is responsible for cellular adhesion [68]. Similarly, *Thy1* codes for a glycoprotein that contains an integrin binding site and plays an important role in cellular adhesion, as well as the migration of immune cells across the endothelium [69]. Furthermore, *Cdh5* is predominantly and almost exclusively expressed in endothelial cells [70], so the reduced endothelial-specific *Cdh5* expression further supports the impact of midostaurin on the endothelium and cervical BSCB after injury. While the 0 mg/kg midostaurin group served as a vehicle control, it is important to note the impact of DMSO on the recovery of animals. Hence, this needs to be considered when making an inference regarding midostaurin-induced transcriptional changes following traumatic cervical SCI.

Changes in inflammatory markers are important verifications in this study. We previously characterized the differential splenic and circulatory inflammatory response following cervical and thoracic injuries [71,72]. These studies indicated that fractalkine and IL-12 are two important cytokines in level-specific responses to injury. In the present study, the reduction of fractalkine further supported the involvement of midostaurin on immune cell recruitment after SCI. Fractalkine (*Cx3cl1*) is a membrane-bound chemokine and, along with its receptor (*Cx3cr1*), is crucial for mediating microglial activity in the spinal cord [73]. Fractalkine is mainly expressed by neurons, whereas its receptor is largely expressed by microglia [74]. Activated endothelia upregulate fractalkine expression and serve as adhesive molecules to mediate reactive immune cell infiltration to the injury epicenter [74]. The genetic knockout of the *Cx3cr1* fractalkine receptor in mice results in enhanced endogenous repair, axon sprouting, and synaptogenesis [74]. Interestingly, previous studies have indicated suppressed fractalkine upregulation in vitro in the presence of a PKC inhibitor, Ro32-0432 [75].

Similarly, the reduced expression of the well-characterized pro-inflammatory markers IL-1α and IL-5 further demonstrated attenuated spinal inflammation. IL-1α is produced by activated immune cells and acts through the IL-1 receptor to initiate a diverse set of inflammatory responses after injury [76]. IL-1α deletion reduces the infiltration of neutrophils and macrophages and improves locomotor recovery [77]. A similar effect was observed following intrathecal administration of anakinra—an IL-1 receptor antagonist [77]. These results were echoed by two intraperitoneal injections of human IL-1 receptor antagonist protein (IL-1RA) into C57BL/6J mice [78]. Furthermore, IL-1α deletion protects oligodendrocytes through the upregulation of the survival factor Tox3 following SCI [77]. The present study demonstrated that the level of IL-1α is elevated after DMSO addition but significantly reduced following midostaurin administration. In contrast to IL-1α, IL-5 is involved in Th-2 responses and allergic eosinophilic responses [79,80]. The allergy aspect and eosinophils are notable here given that SCI may have an autoimmune component and allergies can be argued as having a hyperactive immune component. However, eosinophils generally are not attributed to spinal cord injury etiology [81].

Furthermore, the midostaurin-treated animals exhibited altered levels of MCP-1, IL-13, and IL-4. MCP-1—expressed by the *Ccl2* gene—is a chemokine that mediates the infiltration and migration of macrophages and monocytes through interactions with its receptor, which is expressed by *Ccr2* [82]. The significant reduction in MCP-1 was indicative of the reduced migration of macrophages and monocytes to the site of injury. IL-13 and IL-4 have similar effects on inflammatory processes [83]. However, they exhibited differential patterns in the 25 mg/kg midostaurin cohort compared to the SCI control cohort. Previous studies have demonstrated that the transplantation of IL-13-secreting mesenchymal stem cells to an injured spinal cord significantly enhances functional recovery and decreases lesion size and demyelination [84]. Similarly, the systemic administration of IL-4 exerts immunomodulatory and neuroprotective effects after injury [85].

### 4.2. Neuroprotective Effects of DMSO

DMSO is a clinically approved aprotic solvent that was previously tested for the application of midostaurin in AML studies. Our results indicated that the vehicle solution was well-tolerated after administration, and we did not observe any toxicity or mortality in the 0 mg/kg midostaurin group. DMSO exerts a neuroprotective effect following traumatic SCI, traumatic brain injury, and cerebral infarction [86,87,88]. Mechanistically, DMSO acts as a sodium channel blocker and immunosuppressor that reduces edema and inflammation after injury. It also blocks calcium influx and the activity of NMDA and AMPA channels [88]. Though the impact of DMSO on SCI recovery is modest, this should be noted for future optimization studies. The present study was limited to only infer the neuroprotective role of midostaurin when co-administered with DMSO.

### 4.3. Modulatory Impact of Neuroprotective Treatments

This study suggests the potential for early neuroprotective treatment (midostaurin and DMSO) to restore balanced neural circuit connectivity post-SCI. As GAD67 is involved in the conversion of excitatory glutamate into inhibitory GABA, its alteration suggested a restored excitatory/inhibitory ratio at 8 weeks post-SCI [89]. This was in parallel with restored forelimb/hindlimb base of support, as evidenced by CatWalk^TM^. The base of support is defined by the distance between either forelimb or hindlimb bilateral paw prints during each cycle [90]. The ratio between the forelimb and hindlimb base of support is an indicator of the animal’s balance and interlimb coordination [90]. Recent studies have demonstrated the role of long propriospinal interneurons in regulating forelimb and hindlimb coordination between the cervical and lumbar spinal cord [91]. This was congruent with previous studies demonstrating the role of the inflammatory response on SCI complications, such as neuropathic pain. Furthermore, reduced *Cx3cr1* signaling was found to improve axonal and synaptic plasticity on ventral horn motor neurons [74]. Nevertheless, the results from GAD67 immunohistochemistry could suggest as an interesting future direction to study the modulatory impact of neuroprotective treatments.

### 4.4. Staurosporine-Derived Inhibitors as Therapeutics for Traumatic SCI

Staurosporine is an ATP-competitive general protein kinase inhibitor that is highly permeable across membranes [39,40]. Midostaurin is a derivate of staurosporine with a narrower specificity. Midostaurin gained FDA approval in 2017 for the treatment of acute myeloid leukemia (AML). Enzastaurin (LY-317615) is a further differentiated inhibitor in this family, and it is highly specific for PKC-beta [40]. Enzastaurin has been investigated in phase III clinical trials for diffuse large B cell lymphoma and glioblastoma [92,93]. In an experimental autoimmune encephalomyelitis (EAE) model of multiple sclerosis, the oral administration of enzastaurin significantly reduced vascular permeability and attenuated demyelination and axonal damage [94]. These studies demonstrated the efficacy of staurosporine-derived inhibitors, such as midostaurin, as effective treatment options to control the inflammatory response in CNS disorders. Hence, repurposing staurosporine-derived inhibitors for SCI will have a significant effect on the management of secondary pathology.

## 5. Conclusions

Systemic protein kinase inhibition by a single early midostaurin injection is a promising therapeutic approach to mitigate secondary response after cervical SCI. The results of this study suggest that such a therapeutic approach can improve forelimb and hindlimb coordination during the chronic phase of injury. Future studies on the involvement of individual cell types and signaling cascades, as well as the optimization of dosage and timepoint, could potentially improve the treatment options available to cervical SCI patients.

## Data Availability

The datasets used and/pr analysed during the current study are available from the corresponding author on reasonable request.

## Supplementary Materials

The following are available online at https://www.mdpi.com/article/10.3390/biom11070972/s1, Figure S1: Representative images of the surgical procedure to induce C6/7 laminectomy and C6/7 clip-compression injury, Figure S2: Western blot membranes demonstrating reduced phosphorylation of Signal Transducer and Activator of Transcription 3 (STAT3) and Glycogen Synthase Kinase 3 (GSK3β), Figure S3: Ancillary measurements at 8 weeks post-injury, Figure S4: Representative images of GAD67 and NEUN staining at the dorsal and ventral horns, Table S1: List of antibodies used for western blot and immunohistochemistry, Table S2: Functional classification of inflammatory markers following SCI, Table S3: List of TaqMan primers used for RT-qPCR.
