# Supplementary material for: The Protein Kinase Inhibitor Midostaurin Improves Functional Neurological Recovery and Attenuates Inflammatory Changes Following Traumatic Cervical Spinal Cord Injury
Source: Biomolecules. 2021 Jul 1;11(7):972. doi: 10.3390/biom11070972 (PMC8301989; doi:10.3390/biom11070972)
Supplement: Supplementary file 1 [file biomolecules-11-00972-s001.zip › Supplementary material (2).pdf]

biomolecules

MDPI

Supplementary File

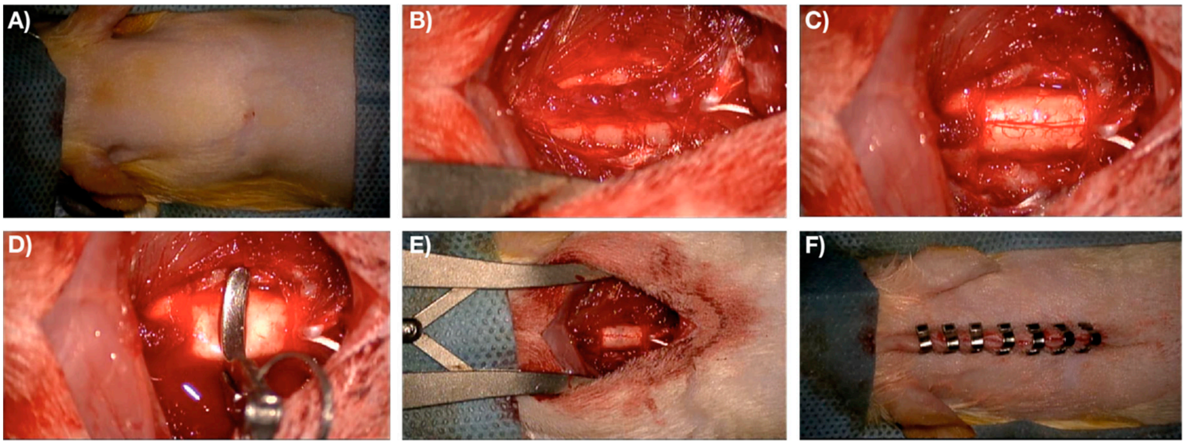

Supplementary Figure S1: Representative images of the surgical procedure to induce C6/7 laminectomy and C6/7 clip-compression injury. A) The wound area was disinfected with Povidone-iodine and ethanol. B) The muscles were incised exposing the laminae. C) The C6/7 laminae was removed using a bone cutter without touching the spinal cord. D) The clip was passed under the spinal cord and released to induce compression-contusion injury. E) The clip was removed, and the local blood was cleared. F) The muscles were sutured and the skin was closed using staples.

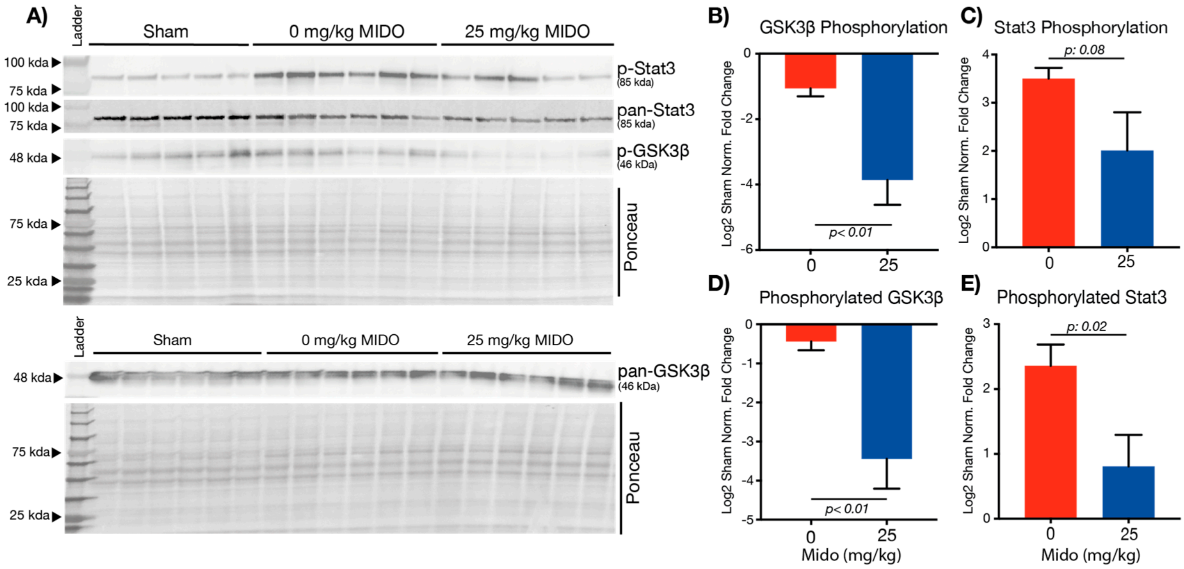

Supplementary Figure S2: Western blot membranes demonstrating reduced phosphorylation of Signal Transducer and Activator of Transcription 3 (STAT3) and Glycogen Synthase Kinase 3 (GSK3β). A) Total protein was visualized via Ponceau S staining. The molecular weights were determined using the Sigma-Aldric BLUeye Prestained Protein Ladder. The membranes were imaged using BioRad Chemidoc™ and analyzed using Bio-Rad Image Lab™ software. The bar graphs illustrate log2 fold change (compared to uninjured shams) band volume. B-C) The two graphs represent the p-GSK3β and p-STAT3 band volumes respectively normalized to Ponceau S total protein staining. D-E) The phosphorylation graphs illustrate the levels of phosphorylated GSK3β and STAT3 normalized to pan-GSK3β and pan-STAT3 levels, respectively.

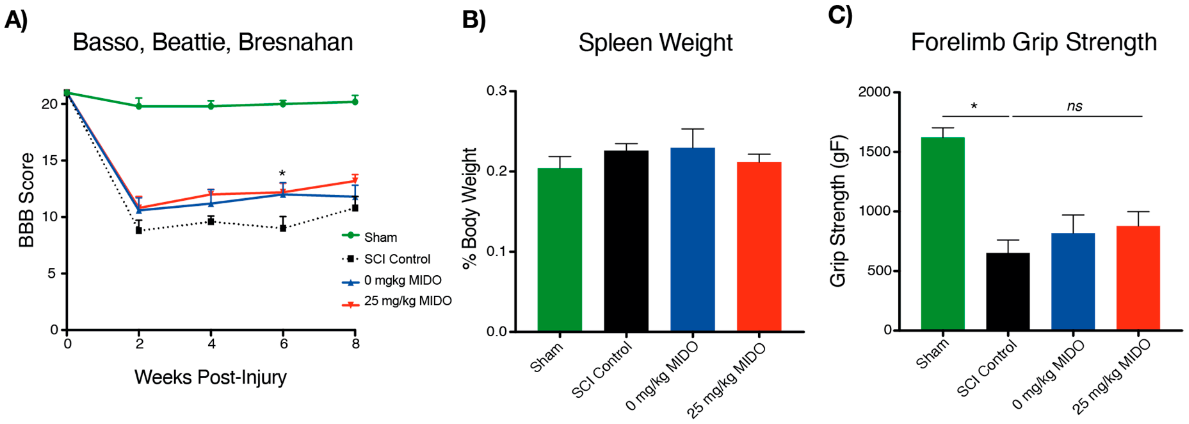

Supplementary Figure S3: Ancillary measurements at 8 weeks post-injury (n=5 per cohort). A-B) Forelimb grip strength and BBB score are significantly reduced after cervical SCI. B) The BBB score is significantly increased in 25mg/kg midostaurin compared to the SCI control. C) Spleen weight was normalized to the time-matched total body weight. No significant change in spleen weight at 8 weeks post-injury.

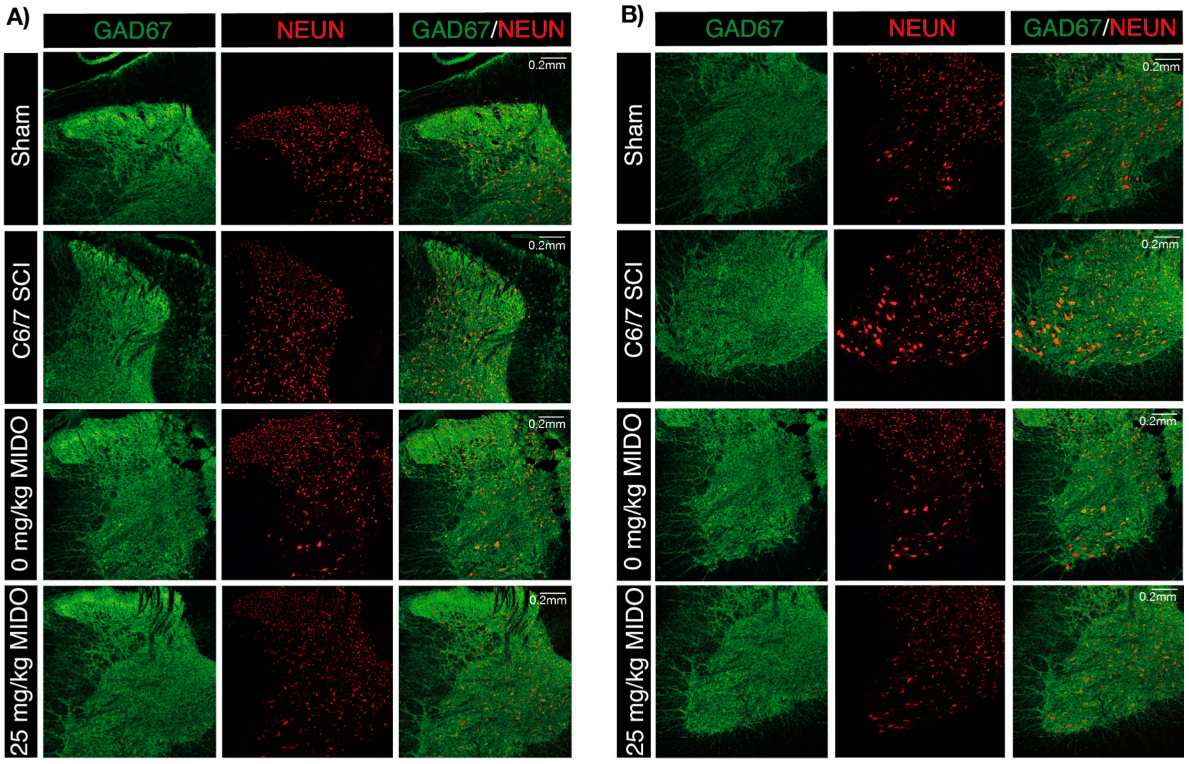

Supplementary Figure S4: Representative images of GAD67 and NEUN staining at the dorsal and ventral horns. A) GAD67 staining (green) and NEUN staining (red) at the dorsal horn. B) GAD67 staining (green) and NEUN staining (red) at the ventral horn.

Supplementary Table S1: List of antibodies used for western blot and immunohistochemistry.

Application	Primary Antibody	Secondary Antibody
**Western blot**	**Anti-Phospho-GSK-3β (Ser9)**<br>Cell Signaling Technology<br>Cat#: 9336S<br>Host species: Rabbit<br>Concentration: 1:250	**Anti-rabbit HRP**<br>Sigma Aldrich<br>Cat#: A0545<br>Host species: Goat<br>Concentration: 1:250
	**Anti-GSK-3β**<br>Cell Signaling Technology<br>Cat#: 9336S<br>Host Species: Rabbit<br>Concentration: 1:250	**Anti-rabbit HRP**<br>Sigma Aldrich<br>Cat#: A0545<br>Host species: Goat<br>Concentration: 1:250
	**Anti-Phospho-Stat3 (Tyr705)**<br>Cell Signaling Technology<br>Cat#: 9131<br>Host species: Rabbit<br>Concentration: 1:250	**Anti-rabbit HRP**<br>Sigma Aldrich<br>Cat#: A0545<br>Host species: Goat<br>Concentration: 1:250
	**Anti-Stat3**<br>Cell Signaling Technology<br>Cat#: 9139S<br>Host species: mouse<br>Concentration: 1:250	**Anti-mouse HRP**<br>Sigma Aldrich<br>Cat#: A3682<br>Host species: Goat<br>Concentration: 1:250
Immunohistochemistry	**Anti-GAD67**<br>Millipore Sigma<br>Cat#: MAB5406<br>Host: Mouse<br>Concentration: 1:500	**Anti-Mouse IgG Alexa Fluor 647**<br>Thermofisher<br>Cat#: A-21235<br>Host: Goat<br>Concentration: 1:500
	**Anti-NeuN**<br>Millipore Sigma<br>Cat#: ABN78<br>Host: Rabbit<br>Concentration: 1:500	**Anti-Rabbit IgG Alexa Fluor 488**<br>Thermofisher<br>Cat#: A-11034<br>Host: Goat<br>Concentration: 1:500

Supplementary Table S2: Functional classification of inflammatory markers following SCI [47].

	Pro-inflammatory	Anti-inflammatory
**Cytokine/Chemokines**	GMCSF<br>IL-1α<br>IL-1β<br>IL-2<br>IL-17A<br>IL-18<br>MCP1<br>IP10<br>Fractalkine<br>LIX<br>MIP2<br>RANTES<br>TNF-α<br>IL-5	GCSF<br>Leptin<br>IL-4<br>IL-10<br>IL-12<br>Eotaxin
**Total counts**	**14**	**6**

Supplementary Table S3: List of TaqMan primers used for RT-qPCR.

	Gene name	Assay ID
**Endogenous control**	*Ppia*	Rn00690933_m1
**Target probes**	*Cxcl12*	Rn00573260_m1
	F11r	Rn00587389_m1
	Itga1	Rn01754645_m1
	Cd99	Rn01441198_m1
	Thy1	Rn00562048_m1
	Cldn3	Rn00581751_s1
	Cdh5	Rn01536708_m1
	Pecam1	Rn01467262_m1
	Itga4	Rn01512798_m1
	Itgb2	Rn01427948_m1
	Itgam	Rn00709342_m1
	Ctnnd1	Rn01486371_m1
	Ctnna2	Rn01421320_m1
	Arhgap5	Rn03036858_s1
	Rhoa	Rn00589172_m1
	Ptk2	Rn00433209_m1
	Pxn	Rn01499294_m1
	Jam2	Rn01498607_m1
	Jam3	Rn01524967_m1
	TJP1	Rn02116071_s1
	Cldn5	Rn01753146_s1
	Ocln	Rn00580064_m1
	Mmp9	Rn00579162_m1
	Lcn2	Rn00590612_m1
	Itgb1	Rn00566727_m1
	Ctnnb1	Rn00584431_g1
**Total Counts**	**27**	**27**
